# Impact of flattening-filter-free techniques on delivery time for lung stereotactic volumetric modulated arc therapy and image quality of concurrent kilovoltage cone-beam computed tomography: a preliminary phantom study

**DOI:** 10.1093/jrr/rrt105

**Published:** 2013-08-26

**Authors:** Keiichi Nakagawa, Akihiro Haga, Akira Sakumi, Hideomi Yamashita, Hiroshi Igaki, Takashi Shiraki, Kuni Ohtomo, Yoshio Iwai, Kiyoshi Yoda

**Affiliations:** 1 University of Tokyo Hospital, Department of Radiology, 7-3-1 Hongo, Bunkyo-ku, Tokyo, Japan; 2 Elekta KK, Research Physics, 3-9-1 Shibaura, Minato-ku, Tokyo, 108-0023, Japan

**Keywords:** FFF, lung, SRT, VMAT, 4D CBCT

To the Editor,

Volumetric modulated arc therapy (VMAT) serves as a means for stereotactic hypofractionated treatment of lung tumors [1]. The present authors proposed an efficient VMAT sequence by restricting leaf speeds of a multi-leaf collimator per gantry rotation angle to below 1 mm/degree, thereby reducing the dose delivery time down to 210 s for a D95 prescription dose of 50 Gy in four fractions [2, 3]. The authors also suggested further reduction of the dose delivery time to below 100 s using flattening-filter-free (FFF) techniques [3].

Recently we evaluated a non-clinical FFF research configuration by adding an FFF filter to our linac system, a Synergy with an MLCi multi-leaf collimator (Elekta AB, Stockholm, Sweden). The FFF filter is made of stainless steel and has a constant thickness of 2 mm for 6- and 10-MV beams. Furthermore, we also tested a non-clinical on-board cone-beam CT (CBCT) system, XVI 5.0 research version, that allows concurrent 4D CBCT imaging during VMAT delivery. It is anticipated that the reduced VMAT delivery time may degrade CBCT image quality due to a much lower number of projection images. The purposes of this study were: (i) to quantify the stereotactic lung VMAT delivery time with FFF, and (ii) to visually inspect CBCT image quality with FFF.

## MATERIALS AND METHODS

A Synergy linac controller, Integrity R1.1 (Elekta AB, Stockholm, Sweden) was additionally installed and used for this non-clinical test, where the dose calibration of 2 cGy/MU was tentatively employed for 6-MV FFF beams because the dosimetry hardware was not fully compatible with FFF. Meanwhile, a non-standard calibration of 2 cGy/MU implies that the resolution of the delivered dose is 0.2 cGy at the maximum dose depth because an Elekta linac accepts an MU resolution of 0.1. A treatment-planning system, Pinnacle version 9 (Philips, Eindhoven, Netherlands), was used for FFF beam modeling with the same dose calibration setting of 2 cGy/MU. Stereotactic VMAT plans with a flattening filter (FF) for three lung tumor patients were arbitrarily selected and corresponding FFF VMAT plans were created under the identical dose prescriptions as follows: planning target volume (PTV) D95 of 50 Gy in four fractions; 50 Gy ≤ dose in PTV ≤ 50.1 Gy; V20, V10 and V5 for each lung ≤ 10%, 20% and 30%, respectively; and dose in spinal cord ≤ 15 Gy. One was a single full arc and the others were partial arc plans with arc angle ranges of − 50° to 180° and − 40° to 180°. The procedures for creating an internal target volume (ITV) and a PTV margin were described in a previous article [3]. During VMAT delivery, dose rates and gantry rotation speeds were varied.

The delivery times and isocenter doses were measured and compared between FF and FFF VMAT plans using a lung phantom including cork layers and a spherical ball made of compressed polyethylene (density 1.0 g/cm^3^, diameter 3 cm) placed inside a water phantom, RT3000-New-Water (R-Tech, Tokyo, Japan). A 0.015 cm^3^ pinpoint ionization chamber (Type 31014, PTW, Freiburg, Germany) was inserted into the ball. The measurement in each plan delivery was repeated four times for averaging, and then a grand average was calculated for the three cases. Subsequently, 4D kilovoltage (kV) CBCT imaging was conducted using an XVI 5.0 research version (Elekta AB, Stockholm, Sweden) during VMAT delivery, together with a motion phantom, QUASAR (Modulus Medical Devices, Ontario, Canada). The motion amplitude was 1 cm in the craniocaudal direction with a cycle time of 3 s, and the image qualities were visually inspected.

## RESULTS AND DISCUSSION

**Fig. 1.**
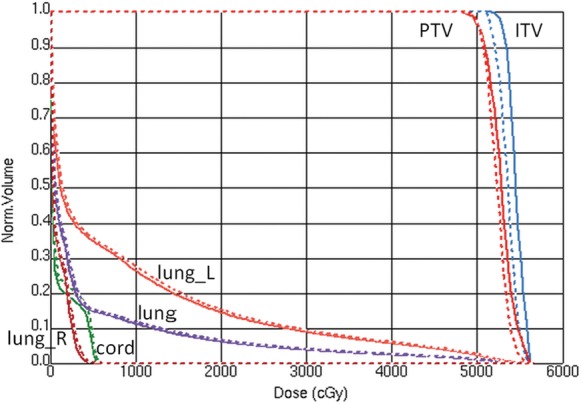
A comparison of dose–volume histograms for a VMAT plan with FF (dotted line) and FFF (solid line). The gantry start and stop angles for a clockwise rotation were −50° and 180°, respectively.

**Fig. 2.**
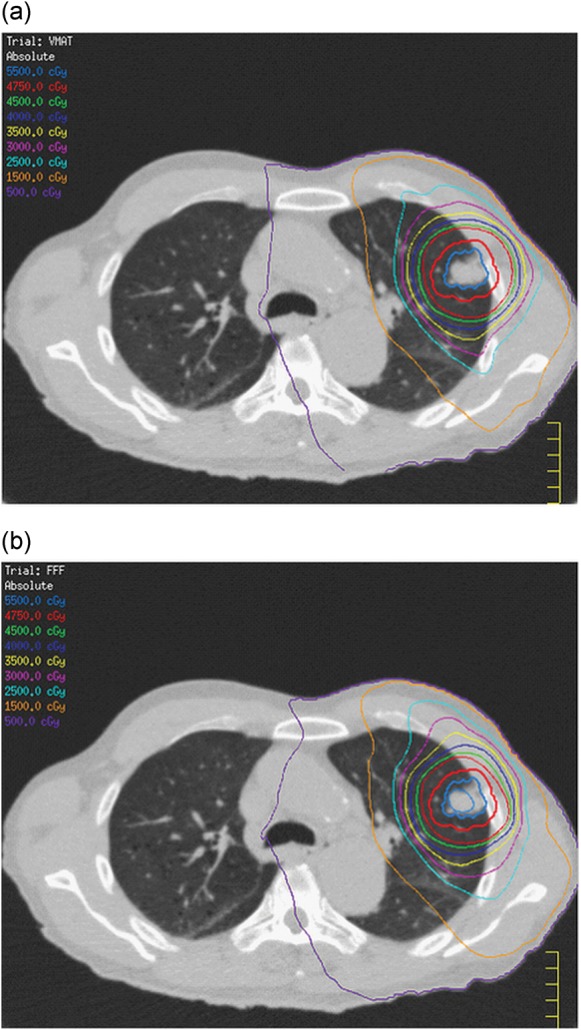
A comparison of calculated dose distributions for the same VMAT plan as shown in Fig. 1 with (a) FF and (b) FFF.

The maximum dose rates for FF and FFF were approximately 550 and 1300 cGy/min, respectively, at the depth of maximum dose. Average beam delivery times for the three cases were 202.7 s (range, 200.5–205.0 s) for FF and 90.0 s (range, 87.5–91.75 s) for FFF. Isocenter dose discrepancies between the plans and the deliveries for the three cases were < 0.96% for FF and < 1.0% for FFF, and the measured doses were always slightly smaller than the calculated doses. Figure 1 shows dose–volume histograms for a partial-arc VMAT plan with FF (dotted line) and FFF (solid line). The dose in ITV was not constrained in the VMAT optimization, and therefore a slightly larger discrepancy was observed. Figure 2 depicts calculated dose distributions for the same VMAT plan with (a) FF and (b) FFF, where ITV (in blue) and PTV (in red) are also shown. It was confirmed that the differences between the FF and FFF deliveries were reasonably small. In this plan, the gantry start and stop angles for a clockwise rotation were − 50° and 180°, respectively.

**Fig. 3.**
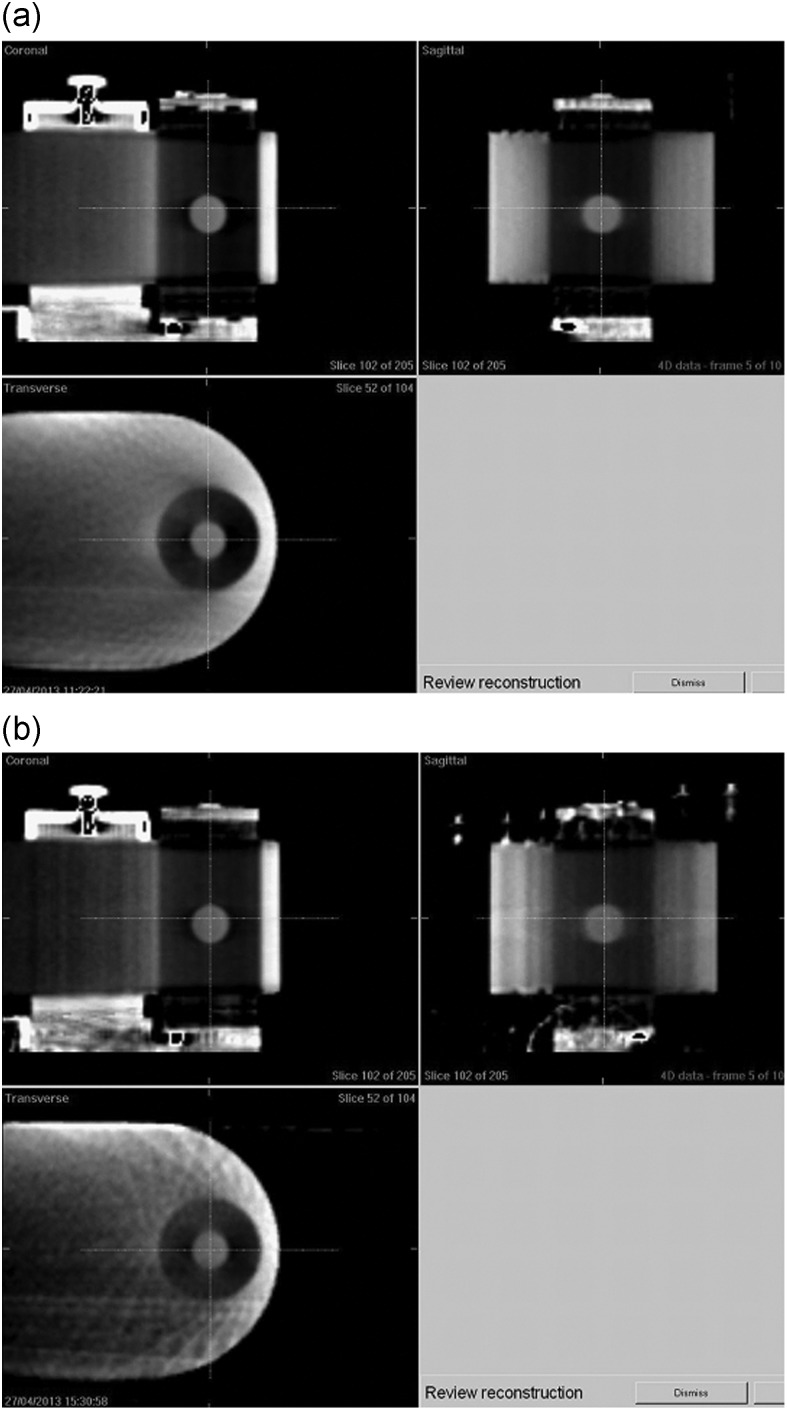
A comparison of inhalation-phase images of concurrent 4D CBCT during VMAT delivery with (a) FF and (b) FFF.

Figure 3 shows inhalation-phase images of concurrent 4D CBCT during VMAT delivery with (a) FF and (b) FFF. The image quality of 4D CBCT with FFF was slightly inferior to that with FF, which can be explained by the fact that the average numbers of projection images for the three cases were 1104 (range, 1093–1116) for FF and 490 (range, 481–500) for FFF. It is anticipated that 4D CBCT with FFF may still be clinically acceptable for 4D tumor registration.

## CONCLUSION

In summary, we have successfully shown an advantage of FFF configuration in terms of dose delivery times for stereotactic lung VMAT. A shorter delivery time ensures more accurate tumor positioning during treatment and possibly greater tumor control. It was also demonstrated that 4D CBCT-based tumor registration is feasible with FFF delivery.

## FUNDING

K.N. received research funding from Elekta.
